# Communicative Adaptations After Laryngectomy: Syntactic Complexity and Gesture Use

**DOI:** 10.1111/1460-6984.70206

**Published:** 2026-02-23

**Authors:** Marise Neijman, Bertus van Rooy, Roland Pfau, Rob J. J. H. van Son, Michiel W. M. van den Brekel

**Affiliations:** ^1^ Department of Head and Neck Oncology and Surgery The Netherlands Cancer Institute Amsterdam The Netherlands; ^2^ Amsterdam Center for Language and Communication (ACLC) University of Amsterdam Amsterdam The Netherlands; ^3^ Department of Oral and Maxillofacial Surgery Amsterdam University Medical Center Amsterdam The Netherlands

**Keywords:** communication, co‐speech gestures, syntactic complexity, total laryngectomy, tracheoesophageal speech

## Abstract

**Background:**

Total laryngectomy (TL) results in the loss of natural voice, requiring alternative speech rehabilitation strategies such as tracheoesophageal speech. While voice and intelligibility outcomes after TL are well studied, less is known about the complexity of spoken language production and the role of co‐speech gestures in this group.

**Aim:**

This study aimed to systematically investigate the complexity and function of spoken language, as well as the use of co‐speech gestures, in TL patients compared with matched controls. The key research question was whether TL patients differ from healthy controls in their syntactic structures and gesture use during structured communicative tasks.

**Methods:**

Forty‐two participants took part: 21 TL patients using tracheoesophageal speech and 21 gender‐, age‐, and education‐matched controls. Participants watched and retold two animated videos and described the “cookie theft” picture. Audio and video recordings were transcribed and analyzed for syntactic complexity (C‐units, subordinate clauses, canonical word order, reduced structures) and typical spoken‐language disfluencies (mazes, fillers, silent pauses). Co‐speech gestures were annotated by type (iconic, metaphoric, deictic, beats, emblem) and function. Word count differences were controlled by normalizing data to 100 words per participant. Outcomes were analyzed using non‐parametric Wilcoxon Rank‐Sum tests with correction for multiple comparisons.

**Results:**

Compared with controls, TL participants produced significantly fewer fillers (*W* = 108, *p* = 0.019) and mazes (W = 109, *p* = 0.019), but significantly more short pauses (W = 356, *p* = 0.001). No significant differences were found between groups for measures of verbal syntactic complexity, except for one omnibus measure (words per C‐unit). Similarly, groups did not differ in gesture frequency, type, or function.

**Conclusions:**

TL patients demonstrated differences in typical spoken‐language disfluencies. However, their use of co‐speech gestures was comparable to that of healthy controls. These findings suggest that while TL impacts spoken language disfluency as part of more careful planning, syntactic complexity is relatively unaffected and gesture use remains intact and may serve as a valuable communicative resource. Clinically, this underscores the importance of recognizing gestures as well as pauses as a compensatory strategy in rehabilitation and counseling following TL.

**WHAT THIS PAPER ADDS:**

*What is already known on this subject*

- Total laryngectomy (TL) affects voice and speech, with rehabilitation often focusing on intelligibility and voice quality.
- Little is known about broader aspects of spoken language complexity and the role of co‐speech gestures in communication after TL.

*What does this study add to existing knowledge*

- TL patients differ from healthy controls in their use of fillers, mazes, and pauses, indicating managing the spoken‐language disfluencies, while leaving syntactic complexity at similar levels than the controls, except for a slight difference in the number of words per C‐unit.
- Despite these changes, TL patients use co‐speech gestures in ways comparable to healthy controls.
- This suggests that gestures remain a stable communicative resource even when spoken language is affected.

*What are the potential or actual clinical implications of this work?*

- Clinicians should recognize the preserved use of gestures as a compensatory strategy for communication after TL.
- Rehabilitation may benefit from explicitly integrating and training disfluencies and gestures into therapy and counseling to support communicative effectiveness.

## 1 Introduction

Effective communication often depends more on conveying the intended message than on grammatical complexity. Nevertheless, speakers frequently adapt their language and nonverbal behavior to facilitate interaction, especially when facing communicative challenges such as language barriers or speech impairments (Altmann and Troche 2011; Plexico et al. 2009). Communication is inherently multimodal, involving both verbal and nonverbal elements, with co‐speech gestures playing an important role in supporting speech, particularly when verbal abilities are compromised (Kendon 1997; Preisig et al. 2018; Sekine et al. 2013; Tetnowski et al. 2023).

### 1.1 Total Laryngectomy

**FIGURE 1:**
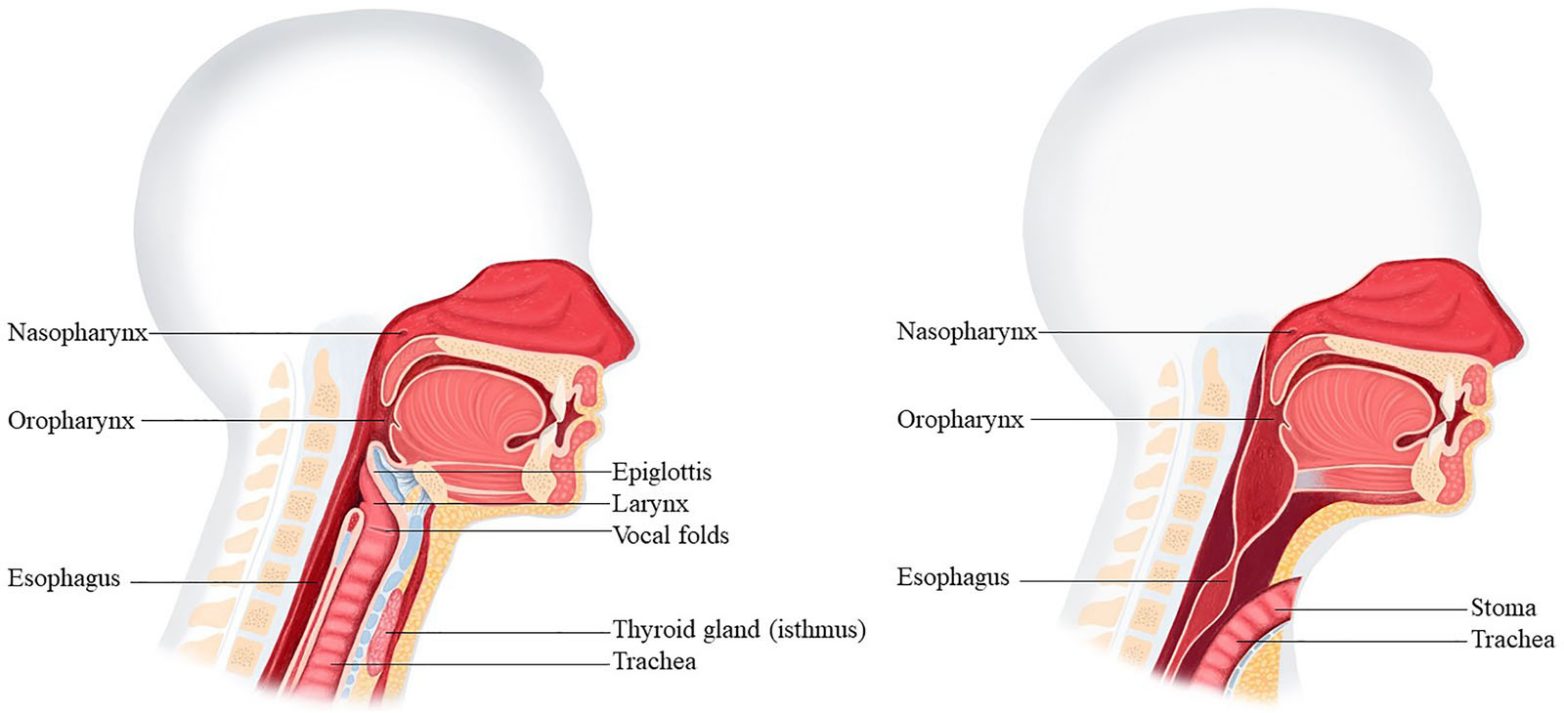
*Anatomy pre‐ and post‐laryngectomy*. Left: healthy anatomy; Right: anatomy after total laryngectomy. These images are used with permission from ATOS Medical, Hörby, Sweden.

A group experiencing primarily physical challenges in speech production are patients who have undergone TL, a surgical procedure removing the larynx, commonly performed to treat advanced laryngeal or hypopharyngeal cancer (Ward and van As‐Brooks 2014). The procedure involves removing the larynx including the vocal folds, permanently separating the upper and lower airways, and creating a tracheostoma at the front of the neck for breathing (see Figure 1). Post‐laryngectomy, patients must adapt to altered speech and breathing, and swallowing in order to communicate effectively (van Sluis et al. 2021).

**FIGURE 2:**
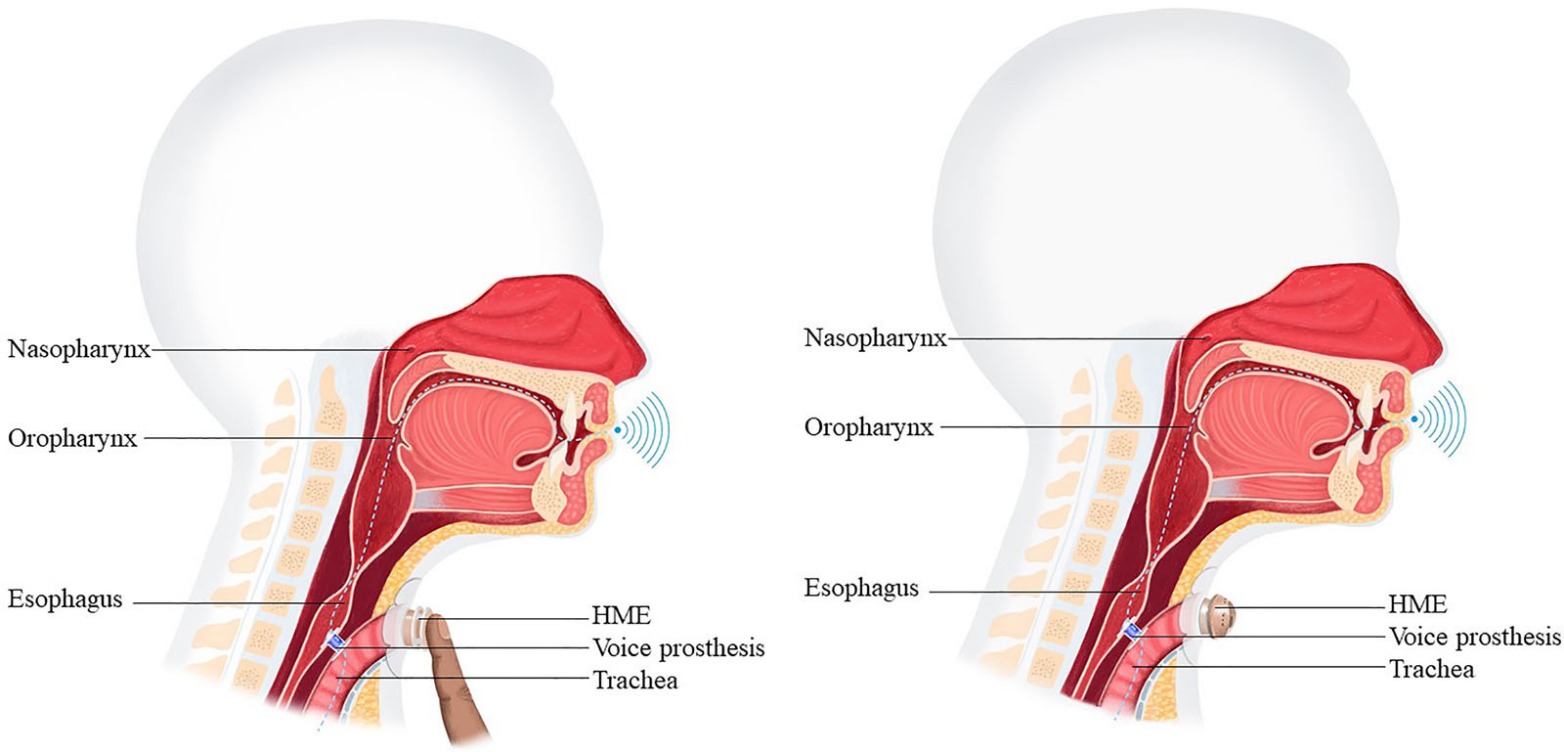
*Physiology of speaking with a voice prosthesis*. Left: manually closing the tracheostoma using the Heat and Moisture Exchanger (HME), Right: speaking using a FreeHands HME. These images are used with permission from ATOS Medical, Hörby, Sweden.

To rehabilitate oral speech, three primary methods are available: the electro‐larynx, esophageal speech, and tracheoesophageal speech (Ward and van As‐Brooks 2014). Tracheoesophageal speech is widely used in Western Europe and is often reported to deliver better acoustic and perceptual voice outcomes than other verbal options (van Sluis et al. 2018; van Weissenbruch et al. 1992; Ward and van As‐Brooks 2014). To achieve tracheoesophageal speech, a voice‐prosthesis (valve) is placed between the trachea and esophagus. When the patient occludes the tracheostoma, the airflow is redirected from the lungs, via the voice prosthesis into the esophagus and new pharynx (neopharynx). The airflow brings the pharyngo‐esophageal segment (PE‐Segment) in vibration, resulting in the new voice sound as seen in Figure 2.

Although tracheoesophageal speech is frequently regarded as the clinical gold standard for voice restoration after TL, it remains substantially different from the natural laryngeal voice in both acoustic properties and the effort required for phonation (Eadie & Doyle, 2005; Searl and Knollhoff 2018; Serra et al. 2015; Tienkamp et al. 2023; van Sluis et al. 2016). Surgical alterations to the anatomy, including the creation of a neoglottis and variations in pharyngo‐esophageal segment closure techniques (e.g., T/Y‐shaped or mucosal closure), affect airflow dynamics and phonation control (van Sluis et al. 2021). The new tracheoesophageal voice typically has a lower pitch, reduced dynamic range, shorter phonation time, and a moist or “bubbly” quality (for acoustic properties of tracheoesophageal speech, see Tienkamp et al. 2023). Speaking often requires considerable effort and coordination, and many patients experience vocal fatigue, particularly in noisy environments (Searl and Knollhoff 2018). Despite intensive speech therapy, voice quality and intelligibility outcomes vary widely, and not all patients regain effective functional communication (van Sluis et al. 2018).

During rehabilitation, patients are taught to adopt a neutral head and body posture, use abdominal breathing, and coordinate inhalation, stoma occlusion, and phonation (Perry 1997). They learn to slow their speech, reduce sentence length, and speak with relaxed neck and shoulder muscles to conserve energy and improve clarity. Two methods of stoma occlusion exist: manual closure of the Heat and Moisture Exchanger (HME) or hands‐free speech using a FreeHands HME (Lorenz et al. 2007), which incorporates a valve that automatically redirects air to the neopharynx. While hands‐free options offer increased convenience and gesture freedom, they also introduce new physical constraints such as increased airflow resistance, the need for precise posture and breathing control, and potential issues with stoma seal or valve responsiveness. Unfortunately, despite speech therapy, not every patient achieves a ‘good’ voice quality or intelligibility using tracheoesophageal speech after a TL (van Sluis et al. 2021).

In addition to physical and functional adjustments, patients who underwent TL face social and psychological challenges (Amechi et al. 2023; Babin et al. 2023; Leemans et al. 2020; Mukoyama et al. 2024; Perry et al. 2015; Sharpe et al. 2019; van Sluis et al. 2020; Wulff et al. 2022). Many feel self‐conscious or anxious about speaking publicly, especially during the early stages of rehabilitation. Concerns about how their new voice is perceived can lead to withdrawal from social interaction. Patients may also require more patience from others, as conversations can demand greater effort from both parties. Since communication is crucial in social relationships, their altered voice may significantly impact both their personal and professional lives.

Previous research on alaryngeal communication has highlighted the importance of both auditory and visual factors in speech perception and communication effectiveness. Listener perceptions vary depending on speech mode and the presence of visual cues (Bridges 1991; Evitts and Gallop 2011; Evitts et al. 2010, Evitts et al. 2021). Nonverbal behaviors such as hand movements and eye gaze also play important roles during face‐to‐face interactions with alaryngeal speakers (Evitts and Gallop 2011; Hartman and Dworkin 1982). These findings underscore that communication after TL is multimodal, involving adaptations in both speech and gestures.

### 1.2 Previous Research

Communication is multi‐modal. It consists of a combination of verbal and nonverbal elements, which makes it reasonable to assume that patients may adapt both their speech and body language to express themselves effectively and manage their communicative situations as successful as possible. To investigate if and how TL influences the use of verbal and nonverbal elements, a pilot study has been conducted (Neijman et al. 2022). In that pilot study, women who underwent a TL were found to use various verbal and non‐verbal techniques to compensate for their functional limitations. For instance, in the verbal aspect of communication, the women appeared to be more certain and careful in planning their utterances and reduced the syntactic complexity of their sentences. Within their non‐verbal communication, the women utilized co‐speech gestures to support their verbal communication, with beat gestures used most often.

Although the earlier pilot study yielded interesting results, caution is warranted when interpreting and generalizing its findings. The study was based on eight interviews focused on the quality of life of women who underwent a laryngectomy, and neither the interviews nor the study design was specifically intended to investigate communication adaptations. Moreover, since the study included only women, it remains unclear whether the findings apply to men, who represent a larger proportion of the TL population. While the interviews were structured, follow‐up questions introduced variability that limits direct comparability. Additionally, the functions of co‐speech gestures were not analyzed. This omission was deliberate, as research on co‐speech gestures in other patient groups, such as individuals with aphasia, indicates that examining gesture functions can provide valuable insights (Akhavan et al. 2018).

Another limitation of the pilot study includes the different control groups that were used, one group specifically for the grammar and another for the co‐speech gestures. For the grammar part, data drawn from existing dataset of recordings were used as the control group. One disadvantage of this dataset was that the speakers were not matched with the women in terms of age, gender or education. For the co‐speech gesture part, the control data consisted of annotated YouTube podcasts with semi‐public figures, who may have undergone media training that could have influenced their speech and gestures use. In other words, neither of the two control groups was directly comparable to the laryngectomized women.

### 1.3 The Current Study

To determine whether the findings of the pilot study are replicable, the current study systematically examines the syntactic complexity and disfluencies of spoken language and the use and function of co‐speech gestures in TL patients compared to a newly collected, matched control group. Unlike the pilot study, all data for patients and controls in the current investigation were generated specifically for this study, ensuring comparability across groups. This approach seeks to enhance the understanding of the impact of TL on adaptions within communication and provides valuable insights for speech therapy counseling and therapy. Understanding why and how TL patients adapt their communication, can help improve their communication possibilities by developing interventions that focuses on the use of gestures to enhance their communication skills and raise awareness of the impact of their disability on their language and grammar.

## 2 Analysis Framework

Before describing the methods in Section 3, it is important to provide an analysis framework for investigating the relevant aspects of verbal communication and co‐speech gestures. This study includes an overview of measures of syntactic complexity and disfluencies in verbal communication, as well as an understanding of how speech and gestures interact to convey meaning. In this context, “communicative adaptation” refers to the adjustments speakers make, both verbal and nonverbal, to facilitate effective communication in the face of physical or functional challenges. These adaptations may include simplifying syntactic structures, modifying lexical choices, or using co‐speech gestures to support or enhance spoken language. Clarifying this relationship provides a foundation for examining the communicative behavior observed in this study.

### 2.1 Syntactic Complexity in Verbal Communication

Syntactic complexity is a key component of verbal adaptation. It reflects both the number of syntactic elements combined within an utterance and the nature of the relationships among them, which together determine the cognitive resources required for production and comprehension (Bulté & Housen, 2012). Although TL constrains physical speech production, mental processing capacity remains intact. Nevertheless, the effort required to plan and produce speech may lead to shorter utterances and syntactic simplification, reflecting an adaptive strategy to convey meaning efficiently.

In this study, syntactic complexity was measured at both a global and a clause‐specific level. Global measures included the mean length per C‐unit, defined as an independent clause with its modifiers, including non‐clausal responses (see Table 1 and Appendix A), and the subordination index, which provide an omnibus indication of syntactic complexity in spoken language (Bulté & Housen, 2012; Hunt 1966). Clause‐level analyses examined the markedness of main clauses (canonical vs. non‐canonical word order) and the structural and functional properties of subordinate clauses (finite vs. non‐finite, adverbial, complement, relative, cleft, parenthetical, or non‐finite complements) (Biber et al. 1999; Hawkins 2007; Miller and Chomsky 1963). In addition, typical spoken language disfluencies, such as ellipsis, mazes, and filled and silent pauses, were analyzed to capture production effort and interactive adaptations in real‐time communication (Biber et al. 1999; Foster et al. 2000). Disfluencies are normal in spoken language, rather than an indication of production failure. Disfluencies support planning for speaking and also perform interactive roles, such as holding the floor while speaking. All measures were normalized per 100 words.

**TABLE 1:** Overview of the parameters of grammatical analysis.

Category	Definition	Example
**C‐unit**	*(a) A main clause with incorporated subordinate clauses (= T‐unit), or*	(a) en ik zie verder niet [wat er gebeurd] and I see further not [what there happened] “and I don't see what else happened there”
	*(b) elliptical variants thereof, or*	(b) Weer de poes. again the cat “The cat again”
	*(c) non‐clausal response tokens such as answers, greetings, affirmations*.	(c) Interviewer: Kunt u het scherm goed zien? “Can you see the screen well?” Participant: Jazeker. “Indeed”
**Subordinate clause**	*A finite or non‐finite clause that performs a particular role relative to the main clause, namely (a) adverbial*,	(a) En voor de rest <fill> eh </fill> <MAZE> de </MAZE> het ziet er leuk uit buiten, als je naar buiten kijkt uit het keukenraam and for the rest <fill> eh </fill> <MAZE> the </MAZE> it looks there fine out outside if you look outside from the kitchen‐window ʻʻAnd the the rest, it looks fine outside, if you look through the kitchen window.
	*(b) complement, or*	(b) Nou ik heb gezien dat een pussycat aan het uitrekenen was op een bord, now I have seen that a pussycat on the calculate was on a board ʻʻNow I have seen that the cat was calculating on a board.
	*(c) relative clause*.	(c) En Tweety pakt een bowlingbal <,> die die door de regenpijp naar beneden gooit and Tweety takes a bowling‐ball that he through the gutter to below throws “And Tweety takes a bowling ball that he throws down through the gutter.”
	*(d) WH‐cleft clauses, a type of emphatic clause, converts some clausal elements into a subordinate wh‐clause that functions like a complement clause, but are labelled separately*.	(d) wat ik me toen verder herinnerde is dat ie dus <fill> eh </fill> de straat op ging what I myself then further reminded is that he thus <fill> eh </fill> the street up went “What I also remind myself of is that he went up the street.”
	*By extension, (e) parenthetical clauses that are inserted into some other clause, and retains a loose relationship to the host clause*,	(e) Die heeft denk ik de kraan vergeten uit te zetten he has think I the tap forgotten out to put “He has, I think, forgotten to close the tap.”
	*and (f) non‐finite verb phrases that complement a non‐auxiliary verb in the main clause*.	(f) en de kinderen zitten langzaam maar zeker de snoeppot leeg te eten and the children sit slowly but surely the sweety‐jar empty to eat “And the children are busy to eat everything in the sweety jar slowly but surely.”
**Canonical word order**	*(a) For Dutch declaratives, subject initial, followed by verb‐second, other arguments and phrasal adverbials, followed by the remaining verbs in the final position*.	(a) Het kuikentje zit wel in een hokje the chicklet sits indeed in a cage “The little chicklet sits in a cate after all.”
	*(b) For interrogatives, either inversion of subject and first verb, followed by the remainder, or*	(b) Gebeurt er buiten nog iets? happens there outside still something “Is something still happening outside?”
	*(c) wh‐element, followed by verb, subject and the remainder*.	(c) Hoe heet dat spel ook alweer? how is called that game also again “What is that game called again?”
	*(d) For imperatives, main verb, followed by non‐subject arguments and adverbials*.	(d) Oh kijk eens aan oh look even at “Oh, look at that.”
**Non‐canonical word orders**	*(a) Adverbial first: clausal/phrasal adverbial, followed by verb‐second, subject, remaining arguments, other phrasal adverbials and final remaining verbs*.	(a) en met een knal slaat hij tegen de gevel and with one bang hits he against the façade “And with a bang, he hits the façade.”
	*(b) Topicalization: any topicalized element in the first position, followed by the verb‐second and the remainder of the clause in the regular word order, without an explicit copy pronoun or adverb in the syntactic position of the topicalized element. [In the actual data set, topicalization almost exclusively affected adverbials that were put in clause‐initial position, but then not followed by the verb‐second element. Rather, then remainder of the clause continued with subject, then verb.]*	(b) helaas het werd verkeerd berekend alas it was incorrectly calculated “Alas, it was calculated incorrectly.”
	*(c) Left‐dislocation: any topicalized element in first position, followed by the subject, then verb‐second and the remainder in regular word order, with an explicit copy pronoun*.	(c) maar dat krukje dat heeft drie poten but that crutch. that has three legs “But that little crutch, it has three legs.”
**Reduced structures**	*(a) Telegram style: omission of some (or all) function words*.	(a) Zoonlief zit in keukenkast cookie pakken son‐love sits in kitchen‐cupboard cookie take “Sonny boy sits in cupboard taking cookie.”
	*(b) Ellipsis: omission of one or more compulsory syntactic constituents (e.g., subject, object, verb) that can be inferred from context*.	(b) Verkeerde liniaal gebruikt wrong ruler used “Used wrong ruler.”
**Filled pause**	*Any vocalization of word‐like duration that forms part of the utterance of a speaker, in Dutch usually transcribed as ‘eh’*.	maar <fill> eh </fill> Tweety die heeft daar een grote bowlingbal but <fill> eh </fill> Tweety he has there a large bowling‐ball “But, uh, Tweety, he has a large bowling ball there.”
**Silent pause**	*(a) Short pause: Any pause of syllable‐like duration during a speech turn used for breathing*.	(a) en dat lukt hem maar niet <,> Want hij is aan het tekenen… and that succeeds him just not <,> because he is on the draw ʻʻAnd he just doesn't succeed <,> because he is still drawing.
	*(b) Long pause: A longer pause used to think, for word retrieval or forming a sentence*.	(b) die kukelt straks ook achterom <,,> Dat was mijn verhaal he tumbles soon also upside‐down <,,> that was my story ʻʻHe will soon tumble down. <,,> That was my story.

The syntactic patterns and spoken language disfluencies analyzed in this study, along with examples, are summarized in Table 1. For a full description of all measures, their definitions, and criteria for classification, see Appendix A.

### 2.2 Co‐Speech Gestures

In addition to verbal production, the participants’ co‐speech gestures were analyzed as part of their communicative adaptations. Gestures are movements of the hands and body that often occur alongside speech, and they can serve both addressee‐oriented and speaker‐oriented functions (Abner et al. 2015; Kendon, 2004; Özyürek, 2012). Following McNeill (1992), Neijman et al. (2022), and Özyürek (2012), gestures were classified into five types: Iconic, Metaphoric, Deictic, Beat, and Emblem. Iconic and Metaphoric gestures, grouped as Representational gestures, visually represent the content of speech, with Iconic gestures reflecting concrete referents or actions and Metaphoric gestures conveying abstract ideas or conceptual metaphors (Lakoff and Johnson 1980). Deictic gestures involve pointing to objects or abstract concepts, Beats are rhythmic movements aligned with speech prosody, and Emblems are conventionalized, culturally specific gestures that can convey meaning independently of speech (Kong et al. 2015; McNeill 1992).

Gestures were analyzed not only for their type but also for their functional role in communication. Some gestures enhance the message for the addressee (e.g., Representational gestures conveying semantic content), while others support the speaker's production processes, such as lexical retrieval or sentence structuring. Certain gestures, like Beats, may serve both the speaker and the addressee by reinforcing prosodic cues and structuring the speech flow. To ensure comprehensiveness, additional categories were included for gestures that could not be identified or for which no specific function could be deduced.

A detailed description of gesture types, functional classifications, and examples is provided in Appendix B, and an overview of types and functions used in this study is presented in Table 2.

**TABLE 2:** Gesture types and functions (Kong et al. 2015; McNeill 1992).

		Gestures that …
**Type**	*Iconic*	model the shape of an object or motion characteristics of an action.
	*Metaphoric*	represent an abstract idea by means of a concrete shape or motion.
	*Deictic*	point towards an object/person or a location in space.
	*Beats*	follow and accentuate the rhythm of speech.
	Emblems	are conventionalized and have (culture‐specific) standardized meanings.
	*Non‐identifiable*	have either an ambiguous connection or lack a direct meaning in relation to the speech content.
**Function**	*Provide substantive information*	provide semantic information that adds to the speech content.
	*Enhance content*	give same semantic content but might help listeners understand the message better, therefore enhancing the communicative intend of the speech.
	*Provide alternative communication*	carry meaning that is not included in the speech content to the point where gesture might even take over speech.
	*Guide and control speech flow*	are synchronized with the pace of speech and reinforce the rhythm of speech.
	*Reinforce intonation or prosody*	emphasize meaning
	*Assist lexical retrieval*	are intended to aid lexical access.
	*Assist sentence re‐construction*	show a modification in sentence structure.
	*No specific function deduced*	do not conform to any of the above or do not seem to have a specific function that relates to surrounding speech.

## 3 Patients and Methods

### 3.1 Participants

Between April 2022 and March 2023, adults (≥ 18 years) who underwent TL were recruited from the institute. Eligible patients were at least six months post‐surgery and any additional treatment (such as (chemo‐) radiation therapy) used tracheoesophageal speech, and had Dutch as their first language. Participants used either manual HME closure or a hands‐free FreeHands HME for tracheoesophageal speech; the type of device was recorded for each participant, as hands‐free devices allow greater freedom for gestural movements. Patients with vision and hearing problems that prevented them from watching two animation videos and describing a picture were excluded.

In total, 21 laryngectomized patients (16 male, 5 female) and 21 matched healthy controls (14 male, 7 female) were included. Controls, matched for age, gender, education, and primarily included the patients' partners or individuals treated at the institute for other cancers. All controls underwent a brief screening/interview by a speech‐language pathologist to confirm they had no difficulties with speech, voice, hearing, or vision. See Table 3 for participant characteristics.

**TABLE 3:** Participant characteristics.

*Laryngectomized individuals*						*Matched healthy Controls*			
#	Gender	Age	Education	Months post‐TL	Speech rehab	#	Gender	Age	Education
1	Male	61	Higher	72	FreeHands	22	Male	63	Higher
2	Male	57	Lower	10	HME	23	Male	64	Secondary
3	Male	88	Higher	41	HME	24	Male	90	Vocational
4	Female	72	Secondary	76	HME	25	Female	74	Secondary
5	Male	70	Higher	15	HME	26	Male	69	Higher
6	Male	65	Vocational	73	HME	27	Male	60	Vocational
7	Male	74	Secondary	67	HME	28	Male	82	Secondary
8	Female	79	Secondary	15	HME	29	Female	73	Secondary
9	Male	73	Higher	39	HME	30	Male	79	Higher
10	Male	53	Vocational	13	HME	31	Male	58	Vocational
11	Male	72	Vocational	94	HME	32	Male	75	Vocational
12	Male	71	Vocational	43	HME	33	Male	77	Vocational
13	Male	61	Higher	52	HME	34	Female	76	Higher
14	Male	78	Vocational	39	HME	35	Male	79	Secondary
15	Male	76	Higher	23	HME	36	Male	76	Secondary
16	Female	68	Vocational	29	FreeHands	37	Female	59	Vocational
17	Male	56	Vocational	23	HME	38	Male	55	Vocational
18	Male	66	Vocational	8	HME	39	Female	61	Vocational
19	Male	67	Higher	65	FreeHands	40	Male	66	Higher
20	Female	72	Higher	213	HME	41	Female	73	Higher
21	Female	73	Higher	12	HME	42	Female	63	Vocational
**Summary**	Males: 16	Mean 69.1 years	Lower: 1	Mean 49 months	FreeHands: 3		Males: 14	Mean 69.2 years	Lower: 0
	Females: 5	(range 53–88)	Secondary: 3	(range 8–213)	HME: 18		Females: 7	(range 55–90)	Secondary: 6
			Vocational: 8						Vocational: 9
			Higher: 9						Higher: 6

### 3.2 Procedure

Before starting the assessment, participants were instructed to sit upright with the HP Pavilion laptop (model 15‐cw1948nd) placed in front of them on the table. Behind the laptop, a SONY ZV‐E10 camera was positioned on a Rollei Mini M1 tripod to record video, focusing on the participant from the knees to the head. Additionally, a SONY IC Recorder ICD‐AX412F was placed next to the laptop to capture audio recordings in .mp3 format.

To ensure systematic data collection, each participant received the three same tasks in the same order. First, the participant was instructed to watch the short animation video “Bowling Ball” (Canary Row Series) and retell the video. The second task was to watch the short animation video “Swing” (Canary Row Series) and retell the video. The final task required participants to describe the Cookie Theft picture (Goodglass et al., 2001) in narrative form. All tasks were chosen to systematically evoke spontaneous speech. Moreover, the two Canary Row videos have been chosen, as they have been shown to be well‐suited for the elicitation of co‐speech gestures (Kita and Özyürek 2003).

### 3.3 Analysis

#### 3.3.1 Verbal Communication

The analysis of verbal communication was performed by transcribing all audio‐recordings. The data collection was performed by the first author (MN), and a research assistant (MV) transcribed all recordings. MN checked all transcriptions for correctness and validity and annotated the fillers, short and long pauses, and reductions. A third researcher (BvR) annotated the communication units (C‐Units), Syntactic Constituents and Mazes, and performed the remainder of the syntactic analyzes (see Table 1 for examples). The analysis of the syntactic patterns was performed using the software program Wordsmith (Scott 2024). The annotated data were then compared between both groups, and a fourth researcher (RvS) conducted the statistical analysis.

#### 3.3.2 Co‐Speech Gestures

The video recordings were collected by MN and annotated by a research assistant (DJ). For each participant, the onset of each co‐speech gesture was noted, and each gesture was classified by type and function according to Table 2. An individual study‐specific Excel scoring sheet was developed and used for each recording to document each gesture's onset time, as well as its type and function.

To classify gestures functionally as serving either as lexical support or prosodic aid, a conservative approach was applied: only gestures clearly serving a lexical function were labeled as such. In ambiguous cases, gestures were categorized as prosodic aids.

Prior to annotation, MN and DJ completed a practice session on a video outside the study database to ensure familiarity with the coding scheme and consistency. MN reviewed 10% of the annotations to assess interrater reliability, which showed excellent agreement (Cohen's *κ* = 0.87). The annotated data were then compared between both groups, with RvS performing the statistical analyses and RP assisting in interpretation and identifying gesture types and functions.

### 3.4 Statistical Analysis

Statistical analyses were conducted using R software (version 4.2.1). Descriptive statistics, including median and range, were calculated to summarize participant characteristics. For the verbal communication, difference in length of spoken text between participants was accounted for by normalizing frequency counts to 100 words. The data were normalized by scaling all measures to a standard of 100 words per participant, ensuring a fair comparison across groups regardless of differences in total word count. The study outcomes (syntactic complexity and co‐speech gestures) were analyzed using non‐parametric Wilcoxon Rank‐Sum test, with the False Discovery Rate (FDR) correction applied to account for multiple testing. Statistical significance was defined as a *p*‐value less than 0.05.

## 4 Results

All 42 participants completed the tasks. No statistically significant differences were found in the total number of words used or in the speech rate (words per minute) between the TL group and the control group (see Table 4).

**TABLE 4:** Statistic results on syntactic complexity.

Syntactic complexity		TL	Controls	Wilcoxon rank‐sum test		
		Median	Median	*W*	*p*‐value	p.FDR
**General measures**	*Total words*	187	255	135	**0.032** ^*^	0.154
	*Speech rate (words per minute)*	156.5	168.7	161	0.134	0.319
**Omnibus syntactic measures**	*Words per C‐Unit*	7.754	8.848	110	**0.008** ^**^	**0.019** ^*^
	*Subordinate clause per C‐Unit*	0.229	0.312	168	0.279	0.359
**Specific syntactic measures**	*Non‐canonical main clauses (proportion of all main clauses)*	0.248	0.245	215	0.906	0.906
	*Non‐finite subordinate clauses (proportion of all subordinate clauses)*	0.236	0.250	197	0.741	0.834
**Spoken language disfluencies**	*Reduced clauses (per C‐unit)*	0.183	0.121	258	0.215	0.322
	*Filled pauses (per 100 words)*	2.559	5.944	108	**0.008** ^**^	**0.019** ^*^
	*Mazes (per 100 words)*	0.523	1.250	109	**0.009** ^**^	**0.019** ^*^
	*Short silent pauses (per 100 words)*	9.942	5.417	356	**0.000** ^***^	**0.001** ^**^
	*Long silent pauses (per 100 words)*	1.011	0.500	260	0.200	0.322

Observations per CU or per 100 words per participant. Group.

TL: Total laryngectomy, Controls: Control speaker. Wilcoxon rank sum test with continuity correction, *p* values and FDR adjusted p values.

Signif. codes: ^***^ 0.001 ^**^ 0.01 ^*^0.05 ns 1.

### 4.1 Verbal Communication

**FIGURE 3:**
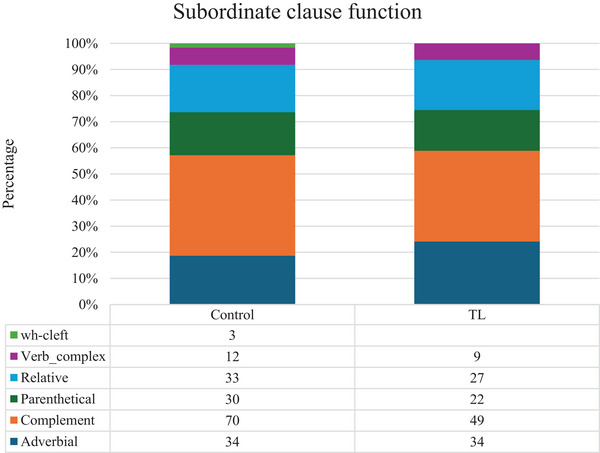
*Subordinate clause function*. Abbreviations: TL, total laryngectomy; Wh‐Cleft, Wh‐cleft clauses; verb_complex, verb complex; relative, relative clause; parenthetical, parenthetical clause; complement, complement clause; adverbial, adverbial clause.

Table 4 provides an overview of the outcomes for syntactic complexity and spoken‐language disfluencies. One of the two omnibus measures of syntactic complexity, the average length of the C‐unit, was significantly shorter for the TL group (*W* = 110, *p* = 0.019), but no significant difference was observed for the number of subordinate clauses per C‐unit. The more specific syntactic measures show no differences, with similar proportions of non‐canonical syntax in the main clauses and similar proportions of non‐finite clauses among the subordinate clauses. The functional distribution of the subordinate clause types is almost identical too, as Figure 3 shows.

The differences between the groups were clearer when the typical disfluencies of spoken language are considered. The TL group used significantly fewer fillers (*W* = 108, *p* = 0.019) and had significantly fewer mazes than the control group (*W* = 109, *p* = 0.019). Conversely, the TL group had significantly more short pauses than the control group (*W* = 356, *p* = 0.001). Reduced clauses and long pauses did not differ significantly between the two groups, though.

### 4.2 Co‐Speech Gestures

Gesture production varied widely within both groups. Table 5 presents the number of used co‐speech gestures per group across the three tasks. The majority of participants in both groups produced less than 10 gestures in total. Three participants from the TL group used 21 or more gestures. Two of them used a non‐FreeHands HME, while one participant with a FreeHands HME was an outlier, producing a total of 41 gestures.

**TABLE 5:** Frequencies of used co‐speech gestures per group.

Number of gestures produced	0	1	2	3	4	5‐9	10‐14	15‐20	21+	Total
Number of TL patients	3 (14%)	1 (5%)	2 (10%)	2 (10%)	3 (14%)	3 (14%)	2 (10%)	2 (10%)	3 (14%)	21 (100%)
Number of controls	5 (24%)	3 (14%)	3 (14%)	1 (5%)	0 (0%)	3 (14%)	3 (14%)	3 (14%)	0 (0%)	21 (100%)

Abbreviations: C, Controls; TL, total laryngectomy.

Table 6 presents the frequencies of the types and functions of co‐speech gestures per group across the three tasks. Regarding the types of co‐speech gestures, Iconic and Beat gestures were the most commonly used in both groups. Similarly, the function of co‐speech gestures was comparable between the groups, with both the TL group and the Control group using their co‐speech gestures primarily to enhance and reinforce intonation. No gestures were found to simultaneously serve both functions.

**TABLE 6:** Frequencies of types and functions of co‐speech gestures.

Type of gesture		Iconic		Beat		Deictic		Metaphoric		Emblem		Total (%)	
Group		TL	C	TL	C	TL	C	TL	C	TL	C	TL	C
**Function of gesture**	*Enhancing*	46	29	1	0	22	6	3	0	0	0	**72 (39)**	**35 (32)**
	*Substantive*	25	23	0	0	7	2	0	0	0	0	**32 (17)**	**25 (23)**
	*Reinforcing intonation*	0	0	55	33	0	0	0	0	0	0	**55 (30)**	**33 (30)**
	*Assisting lexical retrieval*	2	3	1	6	3	2	0	1	0	0	**6 (3)**	**12 (11)**
	*Controlling speech flow*	0	0	20	5	0	0	0	0	0	0	**20 (11)**	**5 (5)**
	*Alternative communication*	0	0	0	0	0	0	0	0	0	1	**0 (0)**	**1 (1)**
	*No specific function*	0	0	0	0	0	0	0	0	0	0	**0 (0)**	**0 (0)**
**Total (%)**		**73 (39)**	**55 (50)**	**77 (42)**	**44 (40)**	**32 (17)**	**10 (9)**	**3 (2)**	**1 (1)**	**0 (0)**	**1 (1)**	**185 (100)**	**110 (100)**

Abbreviations: C, Controls; TL, Total Laryngectomy.

When normalizing the data to 100 words, no statistically significant differences were found in the total number or types of co‐speech gestures between the TL group and the control group (see Table 7).

**TABLE 7:** Non‐parametric Wilcoxon rank‐sum test on co‐speech gestures.

Gestures		TL	Controls	Wilcoxon rank‐sum test		
		Median	Median	*W*	*p*‐value	p.FDR
**General**	*Total gestures*	4	2	309	**0.027** ^*^	0.116
**Types**	*Iconic*	2	2	256	0.372	0.527
	*Beat*	2	1	277	0.147	0.278
	*Deictic*	1	0	284	0.085	0.208
	*Metaphoric*	0	0	232	0.535	0.596
	*Emblem*	0	0	210	0.341	0.527

Observations per 100 words per participant. Wilcoxon Rank‐Sum test with continuity correction, *p* values and FDR adjusted *p* values.

Signif. codes: ^*^0.05 ns 1.

## 5 Discussion

This study systematically explored the syntactic complexity of spoken language, along with the use and function of co‐speech gestures, in a selected group of patients who have undergone TL compared to a matched control group. Results revealed significant differences in a few dimensions of spoken language disfluencies and a single omnibus measure of syntactic complexity. No significant differences were found in the use or type of co‐speech gestures between both groups, meaning that the TL group use a comparable amount and the same type of co‐speech gestures, even though most of them could use only one hand to produce go‐gestures. Overall, the laryngectomy group produced fewer words in total on the tasks, slightly shorter C‐units, fewer verbal disfluencies and more short pauses (for breathing). These findings align with the hypothesis that laryngectomized individuals adapt their multimodal communication strategies, consciously or unconsciously, to their functional abilities. Such adaptation is consistent with broader theories of communicative accommodation and cognitive control, which suggest that speakers adjust their communicative behavior on situational demands and individual capacities (Green and Abutalebi 2013), even though these theories were originally developed in the context of bilingualism.

### 5.1 Verbal Communication

The analysis reveals that only a single omnibus syntactic measure, words per C‐unit, differs significantly between the TL and control group. However, none of the more refined syntactic measures, including the subordination index, reveals significant differences. It appears as if the TL group is simply a little more parsimonious in their word choices to yield slightly shorter C‐units, without making use of different degrees non‐canonical main‐clause syntax or non‐finite subordinate clauses, nor different types of subordination. The fact that their constraint is mainly physical and not cognitive appears to support their ability to use similar syntax than their counterparts in the control group.

A different picture emerges once one considers the typical disfluencies of spoken language. The TL group, possibly due to training during speech therapy, used more frequent short pauses to breathe, while they avoided other typical disfluencies such as filled pauses and mazes. They used silent pauses, which not only gives them opportunity to breathe, but also simultaneously some time to think and plan ahead (Bohnenkamp et al. 2011; Stajner‐Katusić et al. 2006; Stepp et al. 2008), which ties in with the finding that their speech shows significantly fewer disfluencies of the kind that were classified as mazes. Speech timing and pause patterns in the TL group reflect adaptations to altered respiratory and phonatory function after TL (Bohnenkamp 2008; Stepp et al. 2008). Moreover, the TL group also uses fewer filled pauses, which are typical of spoken language conversation, but do require more effortful production of airflow and phonation (Grolman et al. 2008; Searl 2020; Searl and Knollhoff 2018). The combination of more silent pauses and fewer filled pauses may therefore cause trouble for the TL group in regular conversation, since they are less able to signal their intent to hold the floor, while their silent pauses may invite other conversation partners to try to usurp their speech turns. In the context of the present experiment, with a helpful conversation partner that did not try to compete with the speakers for the floor, this eventually did not come to pass, though.

When everything is considered, the TL group is not syntactically constrained in comparison to the control group. They find the physical act of speaking more challenging, and deal with this challenge through pauses and slightly fewer words per C‐units, while compensating through more extensive planning. The resulting speech production is thus of similar diversity and complexity, in contrast to the findings of the pilot study. The differences pertain more to the management of speech turns, a social challenge, and not to the cognitive challenge of conveying the intended message clearly.

### 5.2 Co‐Speech Gestures

Despite producing fewer words overall, the TL group used a comparable number of co‐speech gestures to controls, consistent with prior research showing that gestures are tightly integrated with speech (Neijman et al. 2022). When speech production is less efficient, as in the case of TL patients, gestures may serve as a complementary strategy to support verbal communication. Multi‐modal language production support this by demonstrating the interdependence of speech and gesture during message formulation (Emmorey et al. 2008; Kita and Özyürek 2003; Özyürek, 2012).

Gesture production varied within both groups. Most TL participants (18/21) used a regular HME, leaving only one hand free for gesturing, highlighting their adaptability. Three TL participants used a FreeHands HME, allowing two‐handed gesturing, with one producing an unusually high number of gestures.

Contrary to expectations, TL participants did not rely more heavily on co‐speech gestures as a compensatory strategy, and differences in syntactic complexity were smaller than expected. Both groups primarily used Beat and Iconic gestures similarly, reinforcing speech rhythm and meaning, respectively. Neither group produced Emblems, likely due to the structured and non‐interactive nature of the tasks.

No gestures were found to simultaneously serve both functions (lexical support and prosodic aids). Prior to annotation, the annotators jointly practiced on data outside the study sample to ensure consistent application of decision rules. This approach may have led to an underestimation of lexical gestures but ensured reliable classification.

The equal use of gestures to reinforce intonation across groups was surprising, given the vocal changes in the TL group. This may indicate effective compensatory strategies, possibly relying on deliberate speech pacing or other methods to achieve similar prosodic reinforcement.

These findings align with prior work emphasizing the importance of visual cues in alaryngeal communication (Bridges 1991; Doyle et al. 2023; Evitts et al. 2010, 2011, Evitts et al. 2021; Hartman and Dworkin 1982). Unlike previous studies focusing on natural conversation or audiovisual presentations, our structured tasks show that co‐speech gesture use remains a stable and integrated communicative feature in TL speakers, despite their functional limitations.

### 5.3 Limitations

This study has several limitations that should be considered when interpreting and generalizing the results. Although the design of the study aimed to systematically stimulate spontaneous speech and the use of co‐speech gestures by having participants retell two animated videos and describe a picture, conversational contexts were excluded. This structured approach may limit the applicability of the findings to natural conversational settings.

Furthermore, the comparison in this study was between individuals. The use of syntactic complexity and co‐speech gestures is, however, personal. This study lacked of a pre‐measurement means that within‐subject comparisons before and after surgery were not possible, limiting the ability to assess individual changes in communication strategies over time. As a result, it remains unclear whether observed differences in nonverbal communication and gesture use are due to the effects of surgery or were already present beforehand. A pre‐operative baseline would have allowed for a more precise evaluation of how patients adapt their communication post‐surgery.

In addition, all participants completed the same three tasks in a fixed order: two short animated videos followed by a picture description. This fixed order was chosen to ensure systematic and consistent data collection across participants. Although this approach may raise concerns about potential order effects, such as fatigue or practice effects, the brief nature of the tasks (each video lasting approximately 30 seconds) makes such effects unlikely. Furthermore, since our analyses did not involve comparing performance across the different tasks, possible order effects are not expected to impact the main findings. Nevertheless, future studies could consider counterbalancing task order or employing a crossover design to further control for any order‐related influences.

Moreover, partners were included as control participants to match on age, gender, and education, which helped control for demographic variables. While this ensured demographic comparability, it may introduce potential relationship bias, as partners could share environmental or behavioral traits. This was considered during study design and is acknowledged as a limitation; therefore, results involving controls should be interpreted with due caution. Importantly, the participant and control groups were reasonably balanced for gender (16 M/5F in patients vs. 14 M/7F in controls) and age (mean 69.1 vs. 69.2 years), which reduces the likelihood of confounding effects. Due to the small sample size, statistical testing of potential gender differences in gesture use was not feasible. We acknowledge that previous research suggests that there may be differences in co‐speech gesture use between individuals assigned male versus female gender at birth (Briton et al. 1995; LaFrance 1981; Saucier and Elias 2001; Yang 2010); future studies with larger and more balanced samples could explore these potential gender differences. Additionally, no power analysis was conducted before the study, which may limit the ability to detect smaller effects and impact the reliability of the results. Future studies should include a power analysis to ensure adequate statistical power.

### 5.4 Future Research

Future research could investigate if the communicative adaptions found in this study also occur in a more dynamic and varied communicative settings, such as informal conversations or group discussions, to determine how syntactic complexity and gesture use may differ in less structured contexts. Furthermore, studies should consider incorporating pre‐ and post‐surgical assessments to better understand the communicative changes and their potential impact on daily communication. Developing informative materials for speech and language therapists, future patients undergoing TL, and their loved ones could provide valuable information and help manage expectations in preparation for the surgery. Additionally, speech and language therapists may benefit from the development of targeted therapies to support effective communication adaptations for individuals post‐TL.

## 6 Conclusion

In conclusion, this study demonstrates that, as observed in structured tasks, the TL patients adapt their use of spoken‐language disfluencies to buy time, resulting in minimal difference in syntactic complexity compared to a matched control group. Their use of co‐speech gestures, in both type and frequency, is similarly comparable to controls. Taken together, these findings suggest that although TL affects verbal fluency, gestures remain intact and can be used in rehabilitation and counseling, with training in both disfluencies and gestures helping to support more effective communication.

## Funding

The authors received no financial support for this research.

## Ethical Approval and Consent to Participate

The study was approved by the Institutional Review Board (IRBd21‐226), and all participants provided written informed consent. The study was conducted in accordance with the Declaration of Helsinki.

## Conflicts of Interest

The authors declare no conflicts of interest.

## Data Availability

The data supporting the findings of this study are available from the corresponding author upon reasonable request.

## Appendix A Measures of Syntactic Complexity and Spoken Language Disfluencies

### A.1 Syntactic Complexity

Syntactic complexity is defined as the extent to which syntactic elements combine within a sentence and the relationships among them, determining the processing demands on the language user (Bulté & Housen, 2012, p. 22). Bulté and Housen identify two dimensions of linguistic complexity: (1) the number and nature of the discrete components that an entity consists of, and (2) the number and nature of relationships between these components. More complex structures require greater mental resources to process (Bulté & Housen, 2012, p. 23).

For individuals after total laryngectomy (TL), speech production is physically constrained, but mental processing capacity remains intact. It is hypothesized that, from a mental processing perspective, TL individuals are similar to control speakers. However, physical effort during production may lead to shorter utterances, condensing meaning into fewer words. The need to anticipate these constraints may also impose additional mental demands, potentially contributing to syntactic simplification.

### A.2 Global Measures of Syntactic Complexity

Syntactic complexity can be measured globally or through specific features. Global measures include:

1. T‐unit and C‐unit definitions:
2. **T‐unit**: The minimal terminable unit, defined as exactly one main clause with any attached subordinate clauses (Hunt 1966, p. 737).
3. **C**‐**unit**: Expands the T‐unit to include non‐clausal interactive responses, such as answers to questions, greetings, or exclamations (Loban 1976).
4. **Sub‐clausal units**: Formalized by Foster et al. (2000), these consist of phrases that can be elaborated to full clauses through contextual recovery or minor utterances (Quirk et al. 1985).
5. **Mean length per C‐unit**: Captures the average number of components forming a larger unit, adjusted for spoken language where sentence boundaries are not marked by punctuation.
6. **Subordination index**: Calculated as the number of subordinate clauses per C‐unit. Only subordinate clauses are counted to avoid ambiguity with non‐clausal C‐units (Bulté & Housen, 2012).

### A.3 Clause‐Level Measures

To probe syntactic choices more precisely, analyses at the clause level included:

1. **Main clauses**: Classified as canonical or non‐canonical. Canonical syntax reflects the typical Dutch word order for declaratives, interrogatives, or imperatives. Non‐canonical options include initial adverbials, topicalization, or left‐dislocation, which increase processing complexity. The proportion of C‐units with non‐canonical word order was calculated for TL and control groups.
2. **Subordinate clauses**: Two classifications were performed:
3. **Structural**: Finite vs. non‐finite subordinate clauses, with non‐finite clauses being more complex to process.
4. **Functional**: Adverbial and complement clauses were considered less complex; relative clauses, cleft structures, parenthetical inserts, and non‐finite complements were considered more complex. The ratio of each type was calculated for both groups.

### A.4 Spoken Language Disfluencies

Spoken language contains features that reflect production effort and fluency, all normalized per 100 words:

1. **Ellipsis**: Common in spoken language, ranging from telegram‐style omission of function words to omission of subject or verb that can be recovered from context. TL speakers may produce more elliptical expressions to convey meaning with fewer words and conserve breath.
2. **Mazes**: Non‐propositional disfluencies, such as repetitions, abandoned clauses, and false starts (Biber et al. 1999; Loban 1976). Mazes increase production effort without conveying new information and may be reduced by TL speakers but may still occur under taxing speech conditions.
3. Pauses:
  - **Filled pauses** (e.g., eh in Dutch / uh in English) signal floor‐holding and occur typically at minor syntactic boundaries (Biber et al. 1999, p. 1054).
  - **Silent pauses**: Short pauses are primarily for breathing, while longer pauses are associated with thinking or lexical retrieval. Patients trained in tracheoesophageal speech learn to pause appropriately without disrupting conversation flow.

### A.5 Analytical Rationale

The combination of global and clause‐level measures captures both the number of components and the complexity of relationships within syntactic structures, consistent with theories of linguistic complexity (Bulté & Housen, 2012; Hawkins 2007; Miller and Chomsky 1963). Ellipsis, mazes, and pauses provide additional insight into the constraints imposed by speech production effort in TL speakers.

### A.6 Examples of Analyzed Structures

Table 1 summarizes the syntactic patterns analyzed, including:

- Canonical vs. non‐canonical main clauses
- Finite vs. non‐finite subordinate clauses
- Functional types of subordinate clauses
- Examples of ellipsis, mazes, and pauses

Each example is presented with a word‐for‐word Dutch gloss and idiomatic English translation.

## Appendix B Detailed Co‐Speech Gesture Analysis

### B.1 Theoretical Background

Co‐speech gestures involve movements of the hands and sometimes other parts of the upper body that are often, but not always, produced simultaneously with verbal utterances (Abner et al. 2015). Gestures serve multiple functions in communication, including both cognitive and communicative purposes (Kendon, 2004; Özyürek, 2012). They may assist the speaker in planning, segmenting, or retrieving lexical items, or they may support the addressee in interpreting the spoken message. Evidence that gestures are not purely addressee‐oriented comes from observations of people gesturing while talking on the phone, where the interlocutor cannot see them, suggesting a role in message construction.

Following McNeill (1992) and Özyürek (2012), gestures were classified into five major types: Iconic, Metaphoric, Deictic, Beat, and Emblem. In this classification, Iconic and Metaphoric gestures are jointly referred to as ‘Representational Gestures’, as both convey semantic or conceptual content (Özyürek, 2012).

### B.2 Gesture Types and Definitions

1. **Iconic Gestures**: Visually represent concrete actions, objects, or spatial relations. They bear a motivated relationship to their referent (e.g., moving an index finger in a circular motion while describing a rolling event).
2. **Metaphoric Gestures**: Represent abstract concepts through shape, movement, or orientation (e.g., a backward hand movement over the shoulder to indicate the metaphor PAST IS IN BACK; Lakoff and Johnson 1980).
3. **Deictic Gestures**: Pointing gestures, often with the index finger, directed at concrete or abstract entities in the environment (Kita 2003).
4. **Beat Gestures**: Short, rhythmic, repetitive gestures aligned with the prosody and flow of speech, serving primarily to accentuate or structure utterances.
5. **Emblems**: Conventionalized, culturally specific gestures with stable meaning that can be understood independently of speech (e.g., thumbs‐up = “good” or “perfect”; Kong et al. 2015; McNeill 1992).

### B.3 Gesture Functions

Gestures were also classified according to their functional role in communication:

- **Addressee‐oriented functions**: Enhance or clarify the message for the listener, for example, Representational, Deictic, and Emblem gestures providing semantic or pragmatic information.
- **Speaker‐oriented functions**: Support the speaker in language production, for example, assisting lexical retrieval, sentence construction, or sequencing ideas.
- **Dual‐purpose functions**: Serve both speaker and addressee, for example, Beat gestures that emphasize prosody, rhythm, or discourse segmentation.

Gestures produced without speech or in non‐visible contexts were coded the same as those produced with speech to capture potential speaker‐oriented cognitive functions.

### B.4 Additional Categories

To ensure full coverage:

- **Non‐identifiable gestures**: Movements that could not be reliably classified into the five main types.
- **No specific function deduced**: Gestures for which no clear communicative or cognitive function could be determined.

### B.5 Summary Table

Table 2 provides a structured overview of gesture types, definitions, examples, and functional roles, including the additional categories mentioned above. This table was used to guide coding in the current study and ensures that all observed gestures were systematically categorized.
